# Restorative treatments of dystrophin expression in Duchenne muscular dystrophy: A systematic review

**DOI:** 10.1002/acn3.51149

**Published:** 2020-08-10

**Authors:** Carlos Pascual‐Morena, Iván Cavero‐Redondo, Celia Álvarez‐Bueno, Arthur Eumann Mesas, Diana Pozuelo‐Carrascosa, Vicente Martínez‐Vizcaíno

**Affiliations:** ^1^ Health and Social Research Center Universidad de Castilla – La Mancha Cuenca Spain; ^2^ Universidad Politécnica y Artística del Paraguay Asunción Paraguay; ^3^ Facultad de Ciencias de la Salud Universidad Autónoma de Chile Talca Chile

## Abstract

To evaluate the effect of pharmacological treatments that increase the synthesis of dystrophin in Duchenne muscular dystrophy (DMD). Systematic searches were carried out in MEDLINE, EMBASE, and Web of Science, and in gray literature from inception to December 2019. Clinical trials addressing the effect of restorative treatments of dystrophin expression in children and adolescents with DMD on functional outcomes {(6‐minute walking distance [6MWD], other timed functional tests [TFTs], The North Star Ambulatory Assessment)}, dystrophin expression, cardiorespiratory function, and biochemical tests were included. The DerSimonian‐Laird method was used to calculate the pooled estimates for functional outcomes. Eleven studies were included in the systematic review and five in the meta‐analysis. Eteplirsen showed a significant effect on 6MWD, Δ6MWD = 67.3 m (95% CI: 27.32, 107.28), and Δ6MWD = 151.0 m (95% CI: 36.15, 265.85) at 48 weeks and 3 years, respectively. In the systematic review, analyzing individually the clinical trials using Ataluren and Drisapersen showed a nonsignificant effect on 6MWD. However, the meta‐analysis showed a significant effect on 6MWD for Ataluren and Drisapersen, Δ6MWD = 18.3 m (95% CI: 1.0, 35.5) and Δ6MWD = 21.5 m (95% CI: 4.7, 38.3), respectively. There were no significant differences according to baseline age for Drisapersen. Similarly, the meta‐analysis showed effect in TFT with Ataluren. All drugs induced a partial synthesis of dystrophin, and exon skipping was obtained with Eteplirsen and Drisapersen. Eteplirsen also improved forced vital capacity (Δ%pFVC = 1.8%) and maximal inspiratory pressure (Δ%pMIP = 4.4%). Eteplirsen and Ataluren could modestly reduce disease progression. However, more trials are needed to confirm its efficacy, as well as quality of life and cost‐utility studies.

## Introduction

Duchenne muscular dystrophy (DMD) is a severe X‐linked recessive disease. It manifests in early childhood with muscle weakness, Gowers sign, clumsiness, and difficulty climbing stairs. The disease is progressive, until the loss of ambulatory ability in early adolescence, and other life‐threatening complications such as heart and respiratory failure. This disorder is caused by point mutations, deletions or insertions in the *dystrophin* gene that trigger its truncation and degradation,^1^, ^2^ and occurs once in every 3500–9000 live births.^3^

The only drugs that have shown a delay in the progression of the disease are glucocorticoids, such as Prednisone and Deflazacort.^4^, ^5^ However, in recent years, studies targeted to the partial production of dystrophin have reported promising results, as they have tested therapeutic strategies that can slow the progression of DMD.^6^ Treatments, such as Ataluren, which can force gene reading in the presence of nonsense mutations, or antisense oligonucleotides, restore the reading frame through a splicing mechanism, could have some positive effect, although so far the results and the interpretation of these have been contradictory.^7^, ^8^, ^9^, ^10^ The latter include treatments, such as Eteplirsen and Drisapersen that omit exon 51, Casimersen that omits exon 45, and Viltolarsen and Golodirsen that omit exon 53.^9^, ^10^ Ataluren and Eteplirsen have been conditionally approved by the European Medicines Agency and by the Food and Drugs Agency (FDA), respectively.^11^, ^12^ However, the FDA rejected Ataluren's approval due to serious concerns about the results obtained, since these results were obtained after conducting numerous post hoc statistical and exploratory analyzes. They also suspect that the dose–response pattern reported by one of the trials was quite unlikely.^13^ Also, the FDA has expressed concern about the safety of Golodirsen, and has also asked Sarepta Therapeutics why the development and publication of the Eteplirsen phase III trial its being delayed, as it has been three years since it received conditional approval conditional approval.^14^

Since DMD is an uncommon disease, clinical trials testing the effectiveness of these therapeutic strategies are scarce. A previous systematic review reported an inconclusive effect of exon 51 skipping therapies for patients with DMD on improving timed functional tests (TFTs), that is, 6‐minute walking distance (6MWD), climb 4 stairs, descend 4 stairs, supine to stand, and run 10 m, and the North Star Ambulatory Assessment (NSAA); moreover, significant adverse events were found for Drisapersen.^15^ Because new studies have recently provided data beyond the studies included in the previous review, this novel systematic review aims to update the effect of all pharmacological treatments that increase the synthesis of dystrophin in main functional outcomes (TFTs and NSAA) and other secondary outcomes (dystrophin expression, omission of exon(s), cardiorespiratory function, and biochemical tests) of DMD.

## Materials and Methods

This study was conducted in accordance with the recommendations of the Cochrane Handbook for Systematic Reviews of Interventions^16^ and reported following the Preferred Reporting Items for Systematic Reviews and Meta‐Analyses (PRISMA).^17^ This systematic review was registered in PROSPERO (CRD42018102207), and its protocol published elsewhere.^18^

### Search strategy

MEDLINE (via PubMed), EMBASE, and Web of Science databases were systematically searched. The search was conducted from inception to 31 December 2019. OPEN GRAY, Theseo, Networked digital library of theses and dissertations (NDLTD), Google Scholar, ClinicalTrials.gov, and EudraCT were also searched. The search strategy is detailed in Appendix S1. Trials that used Gentamicin, Ataluren, Eteplirsen, Drisapersen, Casimersen, Golodirsen, or Viltolarsen were potentially eligible. The search was completed by reviewing the references of studies included in the current review.

### Eligibility criteria

The inclusion criteria were as follows: (1) participants: children or adolescents with confirmed DMD, candidates for any drug treatment; (2) design: randomized controlled trials (RCTs), nonrandomized controlled trials (non‐RCTs), and extension studies; (3) interventions: Gentamicin, Ataluren, Eteplirsen, Drisapersen, Golodirsen, Casimersen and/or Viltolarsen treatments; and (4) outcomes: TFTs (6MWD, up 4 stairs, down 4 stairs, run 10 m, supine to stand), NSAA, dystrophin expression, omission of exon(s), cardiorespiratory function, and biochemical changes.

Exclusion criteria were as follows: (1) articles not written in English or Spanish; (2) studies including patients with DMD and other dystrophinopathies in which results were not presented separately; and (3) reports of the same trial when results were duplicated, in which case the most detailed study was included. Excluded trials are described in detail in Table S1.

The literature search was conducted independently by two reviewers (CPM and ICR). Disagreements were solved by consensus or with a third reviewer (V.M.‐V.).

### Data extraction

The following data were extracted in an ad hoc table from the original reports: (1) reference (authors and publication year); (2) type of drug intervention (Gentamicin, Ataluren, Eteplirsen, Drisapersen, Casimersen, Golodirsen, and/or Viltolarsen); (3) country; (4) sample size; (5) groups (intervention/control); (6) age; (7) study design (RCT, non‐RCT, extension study); (8) clinical trial phase (I, II, or III); (9) length of follow‐up; (10) drug dosage; and (11) measured outcomes.

### Risk of bias assessment

Risk of bias of included studies was assessed using the Cochrane Collaboration's tool for assessing risk of bias (RoB2),^19^ which consists of six domains: randomization process, assignment to intervention, adhering to intervention, missing outcome data, measurement of the outcome, and selection of the reported result. Each domain is scored as high risk, some concerns or low risk. Finally, the overall bias is scored as: low risk, when all domains present a low risk score; high risk, when one or more domains present a high risk score; or some concerns, when one or more domains have been scored as some concerns.

The risk of bias assessment was independently conducted by two reviewers (C.P.‐M. and I.C.‐R.). Disagreements and inconsistencies were solved by consensus or with a third reviewer (V.M.‐V.).

### Grading the quality of evidence

The strength of the evidence for main outcomes was assessed using the Grades of Recommendation, Assessment, Development and Evaluation tool (GRADE).^20^ This tool considers three aspects: study design (i.e., RCT, observational studies); factors that decrease the quality of evidence (i.e., risk of bias, inconsistency, indirect evidence, imprecision, and publication bias); factors that increase the quality of evidence (i.e., large effect, possible confounding variables, and dose–response gradient). Finally, the GRADE tool rates the evidence for each intervention and outcome as high, moderate, low, or very low.

### Data synthesis

Forest plots were created to graphically represent the results of each study on the outcomes included (6MWD, TFTs, and NSAA), with the corresponding 95% confidence intervals (95% CI). For outcomes in which there were five or more studies, pooled effects of mean differences and the corresponding 95% CIs were estimated using the DerSimonian‐Laird method.^21^ Positive mean difference values for 6MWD or NSAA indicate a benefit, whereas negative mean difference values for TFTs indicate a benefit. A narrative synthesis of the secondary outcomes was conducted.

Heterogeneity was assessed using the I^2^ statistic. Heterogeneity was considered: not important (I^2^ < 40%), moderate (I^2^ = 30–60%), substantial (I^2^ = 50–90%) or considerable (I^2^> 75%).^14^ The *P*‐value was also considered. A sensitivity analysis (systematic reanalysis by removing each study one at a time) was conducted to assess the robustness of the estimates and detect if any particular study contributed to a large proportion of the heterogeneity. Subgroup analyses were conducted according to the baseline characteristics of the patients (i.e., baseline 6MWD and age at the start of the trial). Publication bias was assessed by visual inspection of a funnel plot, as well as with *Egger´s* test.^22^ A level of <0.10 was used to determine whether publication bias might be present.

Statistical analyses were performed using STATA SE software, version 15 (StataCorp, College Station, TX).

## Results

### Systematic review

**Figure 1:**
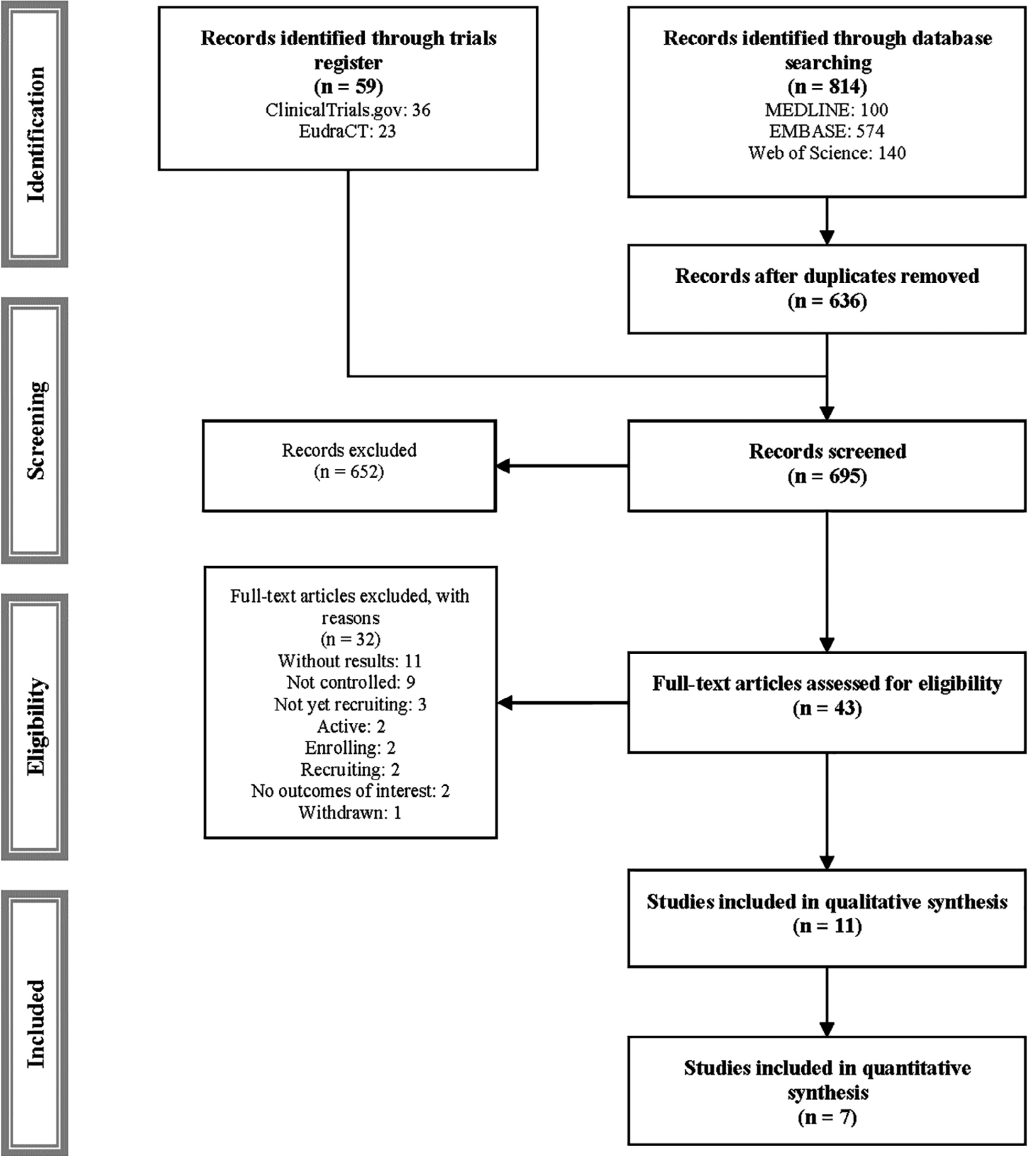
PRISMA flowchart of study selection.

From the 873 studies identified (Fig. 1), 11 met the inclusion criteria.^23^, ^24^, ^25^, ^26^, ^27^, ^28^, ^29^, ^30^, ^31^, ^32^, ^33^ These studies included 917 children and adolescents whose mean ages ranged from 6.9 to 13.1 years old (one study did not report the age of included participants). Four studies analyzed the effect of Eteplirsen, three Drisapersen, one Casimersen, two Ataluren, and one Gentamicin. The sample size of included studies ranged from 12 to 230 (Table 1), and they were conducted in 27 countries: 10 in America, six in Europe, four in Oceania and two in Asia. The studies were published between 2010 and 2019, using the following experimental designs: six RCTs, one cross‐over RCT, one nonrandomized controlled trial and three open extension trials. The drug treatment dosage varied across studies as follows: Gentamicin from 7.5 mg/kg per day to 7.5 mg/kg per week; Ataluren from 40 to 80 mg/kg per day; Eteplirsen from 30 to 50 mg/kg per week; Drisapersen from 3 to 6 mg/kg per week; and Casimersen from 30 mg/kg per day. Finally, the length of the intervention ranged between 24 and 240 weeks.

**Table 1:** Characteristics of included studies.

Reference	Drug	Country	Sample	Groups	Age	Design	Phase	Duration (weeks)	Dosage	Outcomes
Malik et al.^23^	Gentamicin	USA	34	G_1_: 10 G_2_: 8 G_3_: 12 G_4_: 4	NA	NonRCT	I	24	G_1_: 7.5 mg/kg per day, 2 weeks G_2_: control, 2 weeks G_3_: 7.5 mg/kg per week, 6 months G_4_: 7.5 mg/kg/twice week, 6 months	Dystrophin expression Biochemistry Pulmonary function
Bushby et al.^24^	Ataluren	AUS, BEL, CAN, FR, GER, IT, ISR, SPA, SWE, UK, USA	174	G_1_: 57 G_2_: 60 G_c_: 57	G_1_: 8.8 (2.91) G_2_: 8.4 (2.53) G_C_: 8.3 (2.33)	RCT	II	48	G_1_: 40 mg/kg per day G_2_: 80 mg/kg per day G_C_: placebo	TFT Dystrophin expression
McDonald et al.^25^	Ataluren	AUS, BEL, BR, CAN, CH, CR, FR, GER, ISR, IT, PO, SPA, SK, SWE, SWI, TUR, UK, USA	230	G_1_: 115 G_c_: 115	G_1_: 9.0 (7–10) G_C_: 9.0 (8–10)	RCT	III	48	G_1_: 40 mg/kg per day G_C_: placebo	TFT, NSAA
Mendell et al. (2013)^26^	Eteplirsen	USA	12	G_1_: 4 G_2_: 4 G_c_: 4	G_1_: 9.3 (0.5) G_2_: 8.5 (1.29) G_C_: 8.5 (1.73)	RCT + CR	II	48 (24 + 24)	G_1_: 30 mg/kg per week G_2_: 50 mg/kg per week G_C_: placebo	TFT Dystrophin expression Omission exon 51
Mendell et al.^27^	Eteplirsen	IT, USA	99	G_1_: 6 G_2_: 6 G_H_: 87	G_1+2_: 9.3 (1.22)	OET	II	144	G_1_: 30 mg/kg per week G_2_: 50 mg/kg per week G_H_: matched HC	TFT Pulmonary function
Kinane et al.^28^	Eteplirsen	USA	46	G_1_: 6 G_2_: 6 G_H_: 34	G_1+2_: 13.1 (1.08) G_H_: 10.1 (2.22)	OET	NA	240	G_1_: 30 mg/kg per week G_2_: 50 mg/kg per week G_H_: matched HC	Pulmonary function
Charleston et al.^29^	Eteplirsen	USA	24	G_1_:11 G_c_:13	NA	OET	NA	180	G_1_: 30 or 50 mg/kg per week G_c_: untreated	Dystrophin expression Omision exon 51
Voit et al.^30^	Drisapersen	AUS, BEL, FR, GER, ISR, NET, SPA, TUR, UK	53	G_1_: 18 G_2_: 17 G_c_: 18	G_1_: 7.2 (1.7) G_2_: 7.7 (1.5) G_C_: 6.9 (1.2)	RCT	II	48	G_1_: 6 mg/kg per week G_2_: 6 mg/kg per week, fortnightly G_C_: placebo	TFT, NSAA Dystrophin expression Omision exon 51 Biochemistry
Goemans et al.^31^	Drisapersen	ARG, BEL, BR, CAN, CH, CR, DEN, FR, GER, IT, JAP, SK, NET, NOR, PO, RU, SPA, TAI, TUR	186	G_1_: 125 G_C_: 61	G_1_: 8.3 (2.4) G_C_: 8.0 (2.4)	RCT	III	48	G_1_: 6 mg/kg per week G_C_: placebo	TFT, NSAA Dystrophin expression Omision exon 51 Biochemistry
McDonald et al.^32^	Drisapersen	USA	51	G_1_: 17 G_2_: 18 G_c_: 16	G_1_: 7.8 (1.9) G_2_: 7.6 (2.6) G_c_: 8.0 (1.8)	RCT	II	48	G_1_: 3 mg/kg per week G_2_: 6 mg/kg per week G_c_: placebo	TFT, NSAA Omision exon 51 Biochemistry
NCT02500381 (2019)^33^	Casimersen	AUS, BEL, BUL, CAN, CR, FR, GER, HUN, ISR, IT, PO, SPA, SWE, UK, USA	43	G_1_:27 G_c_:16	NA	RCT	III	48	G_1_: 30 mg/kg per week G_c_: placebo	Dystrophin expression

Non‐RCT, Non‐Randomized Controlled Trial; RCT, Randomized Controlled Trial; CR, Crossover trial; OET, Open Extension Trial; 6MWD, 6‐minute walking distance; TFT, Timed functional test; NSAA, North Star Ambulatory Assessment.

#### Main outcomes

**Figure 2:**
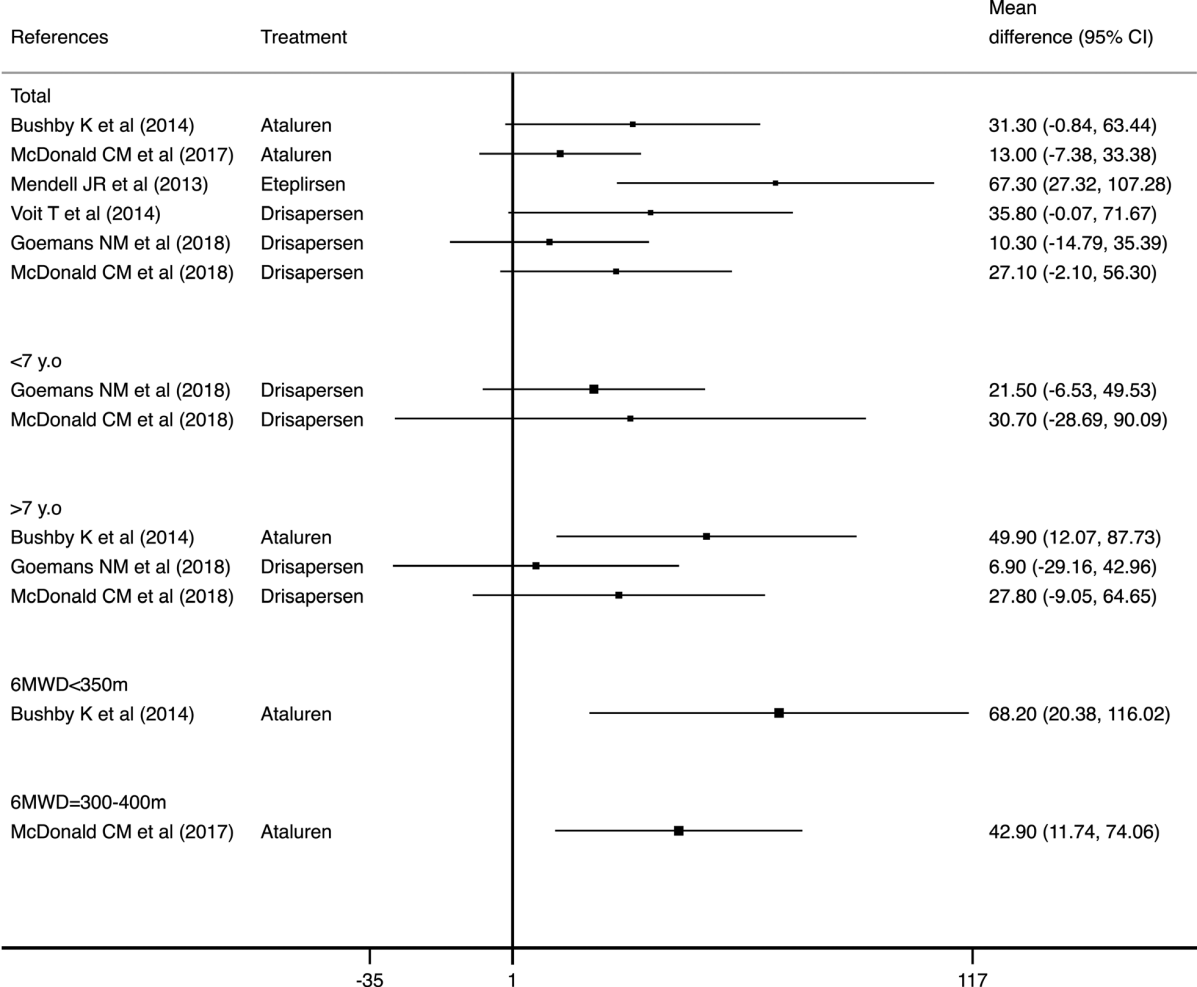
Effect of Ataluren or antisense oligonucleotides versus control on 6MWD, measured in meters (mean difference, 95% CI). Positive effect indicates effect in favor of the intervention group, whereas negative effect indicates effect in favor of the control group. The patients included in the subgroup analysis (i.e., <7 years old, >7 years old, 6MWD <350 m, 6MWD = 300–400 m) were all from the samples of the studies included in the “total” group.

Seven studies evaluated the effect of treatments on TFTs and NSAA. Eteplirsen showed a significant positive effect on 6MWD, with a mean difference of 67.3 and 151.0 m, at 48 weeks and 3 years, respectively.^26^, ^27^ Furthermore, Ataluren (between 13.0 and 31.3 m) and Drisapersen (between 10.3 and 27.1 m) showed nonsignificant effects in 6MWD at 48 weeks.^24^, ^25^ Based on the 6MWD baseline, Ataluren showed an inconsistent effect. In one study Ataluren showed more effect when the 6MWD baseline was <350 m (Δ6MWD = 68.2 m), whereas another study had more effect when the 6MWD baseline was 300–400 m (Δ6MWD = 42.9 m)^25^ (Table 2 and Fig. 2).

**Table 2:** Main outcomes: TFT – 6‐minute walking distance.

Reference	Drug	Dosage	Control group	Subgroup	6MWD	
					$\Delta \bar{x}$ (m)	SE (m)
Bushby et al.^24^	Ataluren	40 mg/kg per day	Placebo	Total	31.3	16.4
		40 mg/kg per day	Placebo	6MWD <350 m	68.2	24.4
		40 mg/kg per day	Placebo	>7 y.o.	49.9	19.3
		80 mg/kg per day	Placebo	Total	−0.7	–
McDonald et al.^25^	Ataluren	40 mg/kg per day	Placebo	Total	13.0	10.4
		40 mg/kg per day	Placebo	6MWD = 300–400 m	42.9	15.9
Mendell et al.^26^	Eteplirsen	30/50 mg/kg per week	Placebo/delayed	Total	67.3	20.4
Mendell et al.^27^	Eteplirsen	30/50 mg/kg per week	Historical cohort	Total	151.0	58.6
Voit et al.^30^	Drisapersen	6 mg/kg per week	Placebo	Total	35.8	18.3
Goemans et al.^31^	Drisapersen	3 mg/kg per week	Placebo	Total	−8.9	15.4
		6 mg/kg per week	Placebo	Total	10.3	12.8
		6 mg/kg per week	Placebo	<7 y.o.	21.5	14.3
		6 mg/kg per week	Placebo	>7 y.o.	6.9	18.4
McDonald et al.^32^	Drisapersen	6 mg/kg per week	Placebo	Total	27.1	14.9
		6 mg/kg per week	Placebo	<7 y.o.	30.7	30.3
		6 mg/kg per week	Placebo	>7 y.o.	27.8	18.8

Results expressed as $\Delta \bar{x}$ (mean difference) and SE (standard error). Positive effect indicates effect in favor of the intervention group, whereas negative effect indicates effect in favor of the control group.

**Figure 3:**
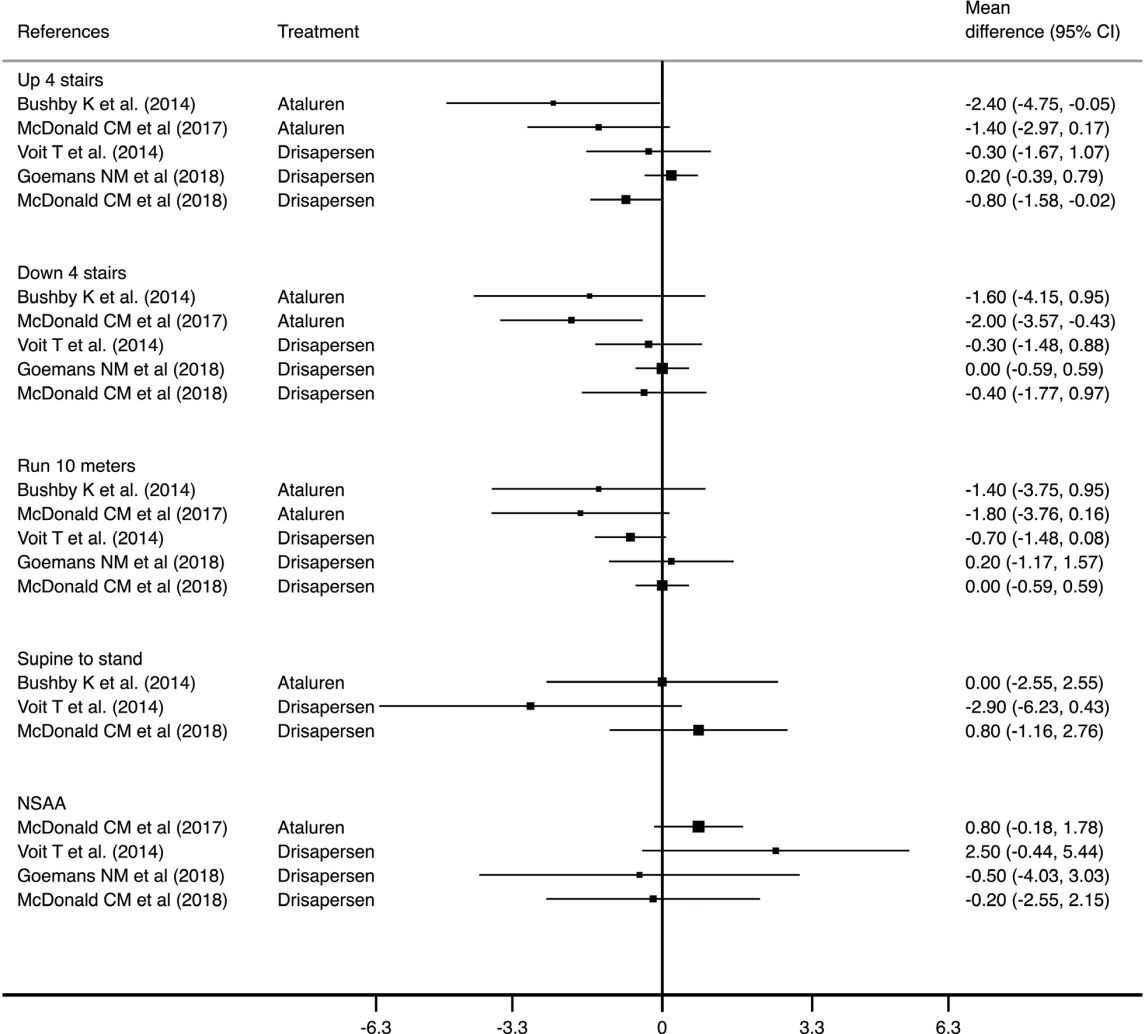
Effect of Ataluren or antisense oligonucleotides versus control on others Timed Functional Test, measured in seconds (mean difference, 95% CI); effect of Ataluren or antisense oligonucleotides versus control on North Star Ambulatory Assessment, measured in score (mean difference, 95% CI). For TFTs, negative effect indicates effect in favor of the intervention group, whereas positive effect indicates effect in favor of the control group. For NSAA, positive effect indicates effect in favor of the intervention group, whereas negative effect indicates effect in favor of the control group.

For Eteplirsen, the authors did not report data from the other TFTs and NSAA. Ataluren showed a moderate decrease in time on TFTs. For TFT‐“climb 4 stairs” between −2.4 and −1.4 sec; for TFT‐“descend 4 stairs” between −1.6 and −2.0 sec; and for TFT‐ “run 10 m” between −1.4 and −1.8 sec. ^24^, ^25^ No effect on the TFT‐“supine to stand” test was observed. Drisapersen did not show consistent results in all TFTs: for TFT‐“climb 4 stairs” between 0.2 and −0.8 sec; for TFT‐“descend 4 stairs” between 0.0 and −0.4 sec; for TFT‐“run 10 m” between 0.2 and −0.7 sec; and for TFT‐“supine to stand” between 0.8 and −2.9 sec (Fig. 3).^30^, ^31^, ^32^ Finally, Ataluren showed an positive effect in NSAA, whereas Drisapersen also showed inconsistent results (Table 3 and Fig. 3).

**Table 3:** Main outcomes: other timed functional test and North Star Ambulatory Assessment.

Reference	Drug	Dosage	Subgroup	Up 4 stairs		Down 4 stairs		Run 10 m		Supine to stand		NSAA	
				$\Delta \bar{x}$ (sec)	SE (sec)	$\Delta \bar{x}$ (sec)	SE (sec)	$\Delta \bar{x}$ (sec)	SE (sec)	$\Delta \bar{x}$ (sec)	SE (sec)	$\Delta \bar{x}$	SE
Bushby et al.^24^	Ataluren	40 mg/kg per day	Total	−2.4	1.2	−1.6	1.3	−1.4	1.2	0.0	1.3	–	–
McDonald et al.^25^	Ataluren	40 mg/kg per day	Total	−1.4	0.8	−2.0	0.8	−1.8	1.0	–	–	0.8	0.5
Voit et al.^30^	Drisapersen	6 mg/kg per week	Total	−0.3	0.7	−0.3	0.6	−0.7	0.4	−2.9	1.7	2.5	1.5
Goemans et al.^31^	Drisapersen	6 mg/kg per week	Total	0.2	0.3	−0.0	0.3	0.2	0.7	–	–	−0.5	1.8
McDonald et al.^32^	Drisapersen	6 mg/kg per week	Total	−0.8	0.4	−0.4	0.7	0.0	0.3	0.8	1.0	−0.2	1.2

Results expressed as $\Delta \bar{x}$ (mean difference) and SE (standard error). For TFT, negative effect indicates effect in favor of the intervention group, whereas positive effect indicates effect in favor of the control group. For NSAA, positive effect indicates effect in favor of the intervention group, whereas negative effect indicates effect in favor of the control group.

#### Secondary outcomes

Ten of the included studies assessed the effect of treatments on secondary outcomes (dystrophin expression, exon skipping, cardiorespiratory function, or biochemical changes). Gentamicin,^23^ Ataluren,^24^ Eteplirsen,^26^, ^29^ and Casimersen^33^ produced an increase in dystrophin expression; however, Drisapersen showed contradictory effects.^30^, ^31^, ^32^ Eteplirsen and Drisapersen caused the omission of exon 51^26^, ^29^, ^30^, ^32^ and Casimersen the omission of exon 45.^33^ For cardiorespiratory function, Eteplirsen reduced the decline in forced vital capacity (FVC) and improved maximal inspiratory pressure (MIP).^27^, ^28^ Drisapersen showed no effect on cardiorespiratory function. Finally, for biochemical changes, Drisapersen decreased creatine kinase (CK) and lactate dehydrogenase (LDH) levels (Table 4).^30^, ^31^, ^32^

**Table 4:** Secondary outcomes

Reference	Drug	Histological changes	Omission of exon	Cardiorespiratory function	Biochemical tests
Malik et al.^23^	Gentamicin	↑ Dystrophin expression in 9 patients, 3 in therapeutic range: Patient 6: 13.0% (WB) Patient 10: 14.0% (WB) Patient 12: 13.5% (WB)	NA	↑ FVC: 1.74–1.84 L/sec (pre‐post)	↓ CK in 14 days: 11,320–5429 U/L (treatment) versus 14,434–14,448 U/L (control). ↓ CK in 6 months: 9851–5316 U/L (pre‐post)
Bushby et al.^24^	Ataluren	↑ Dystrophin expression: 2.8% with 40 mg/kg versus 0.09% with placebo (dystrophin/spectrin)	NA	NA	NA
Mendell et al.^26^	Eteplirsen	↑ Dystrophin expression (WB) ↑ Positive fibers: 47.3% (30–50 mg/kg) versus 37.7% (placebo‐delayed)	↑ Omission exon 51 (RT‐PCR)	NA	NA
Mendell et al.^27^	Eteplirsen	NA	NA	Stable respiratory function (pre‐post): %pMIP = −2.2% %pMEP = −5.0% %pFVC = −9.4%	NA
Kinane et al.^28^	Eteplirsen	NA	NA	Improve pulmonary function: %pFVC: −2.3%/year versus −4.1%/year expected %pMEP = −2.6%/year versus −2.7%/year expected %pMIP = 0.6%/year versus −3.8%/year expected	NA
Charleston et al.^29^	Eteplirsen	↑ Dystrophin expression: 11.6‐fold increase, treatment versus control (WB) ↑ Positive fibers: 7.4‐fold increase, treatment versus control (PDPF)	↑ Omission exon 51: 100% of patients	NA	NA
Voit et al.^30^	Drisapersen	↑ Dystrophin expression: 29% of patients (WB)	↑ Omission exon 51: 11.8% of patients	No changes in pulmonary function (mean difference): FVC: −0.04 L, *P* = ns	↓ CK: −1736 U/L (*P* = 0.303)
Goemans et al.^31^	Drisapersen	NA	NA	No changes in FVC, FEV1, PF, PCF	↓ CK: −5273.5 U/L treatment versus −1228.5 U/L placebo (*P* < 0.001) ↓ LDH: −375.3 U/L treatment versus −119.2 U/L placebo (*P* < 0.001)
McDonald et al.^32^	Drisapersen	Not change in dystrophin expression (IF and WB)	↑ Omission exon 51 (RT‐PCR)	No changes in pulmonary function (mean difference): FVC: −0.01 L, *P* = ns FEV_1_: −0.03 L, *P* = ns PEF: −12.30 L/min, *P* = ns PCF: 1.5 L/min, *P* = ns	=CK: −2287.2 U/L treatment versus −2790.7 U/L placebo =LDH: −239.5 U/L treatment versus −246.2 placebo
NCT02500381 (2019)^33^	Casimersen	↑ Dystrophin expression: 0.925–1.736% (WB) (pre‐post)	↑ Omission exon 45: 100% of patients. R Spearman for omission exon – dystrophin expression = 0.635	NA	NA

WB, Western blot; PDPF, percent dystrophin‐positive fibers; IF, immunofluorescence; RT‐PCR, Reverse transcription polymerase chain reaction; FVC, forced vital capacity; MIP, maximal inspiratory pressure, MEP, maximal expiratory pressure; FEV1, forced expiratory volume in 1 second; PEF, peak expiratory flow; PCF, peak cough flow; CK, creatine Kinase; LDH, lactate dehydrogenase.

### Risk of bias assessment

**Figure 4:**
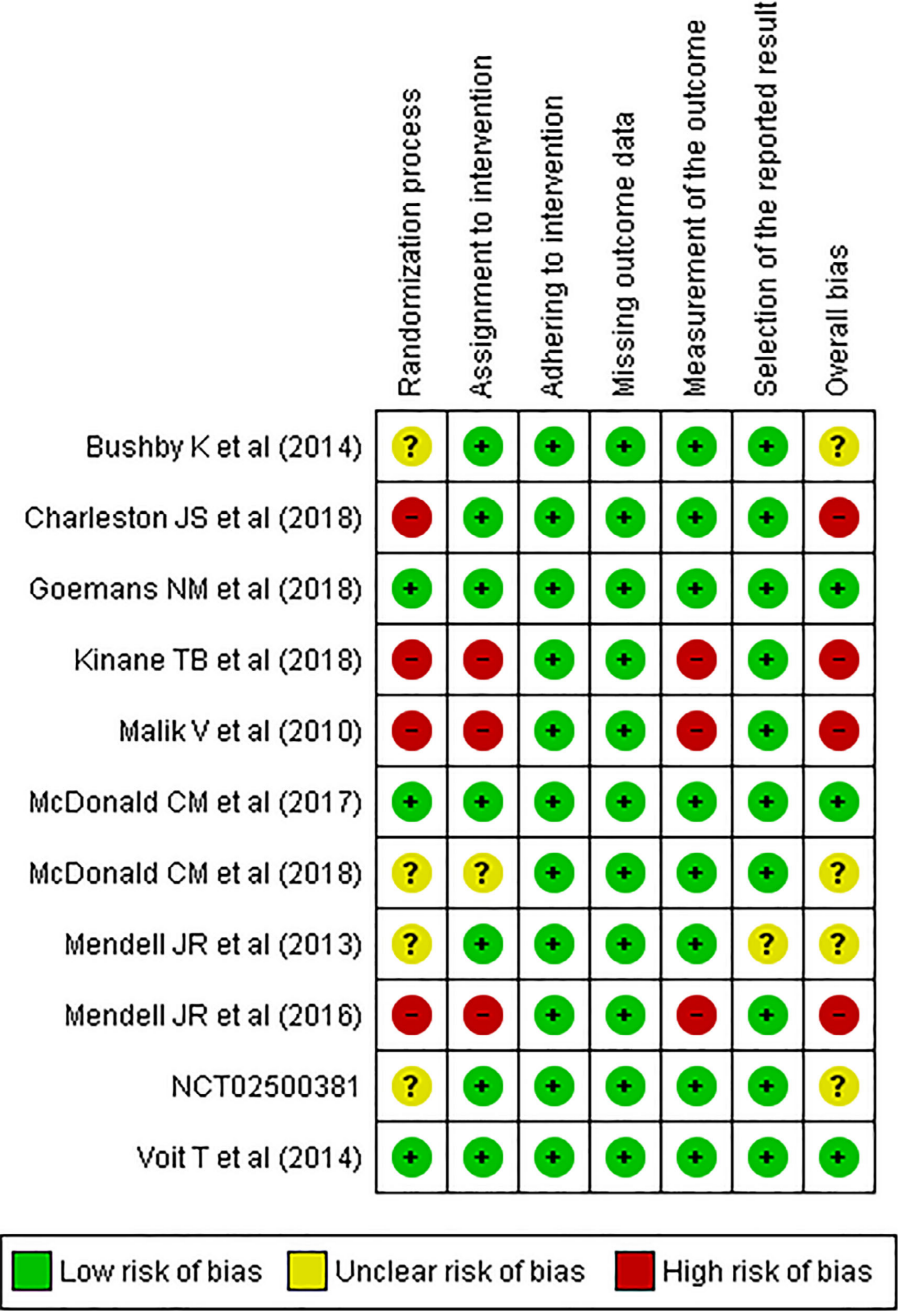
Risk of bias summary.

**Figure 5:**
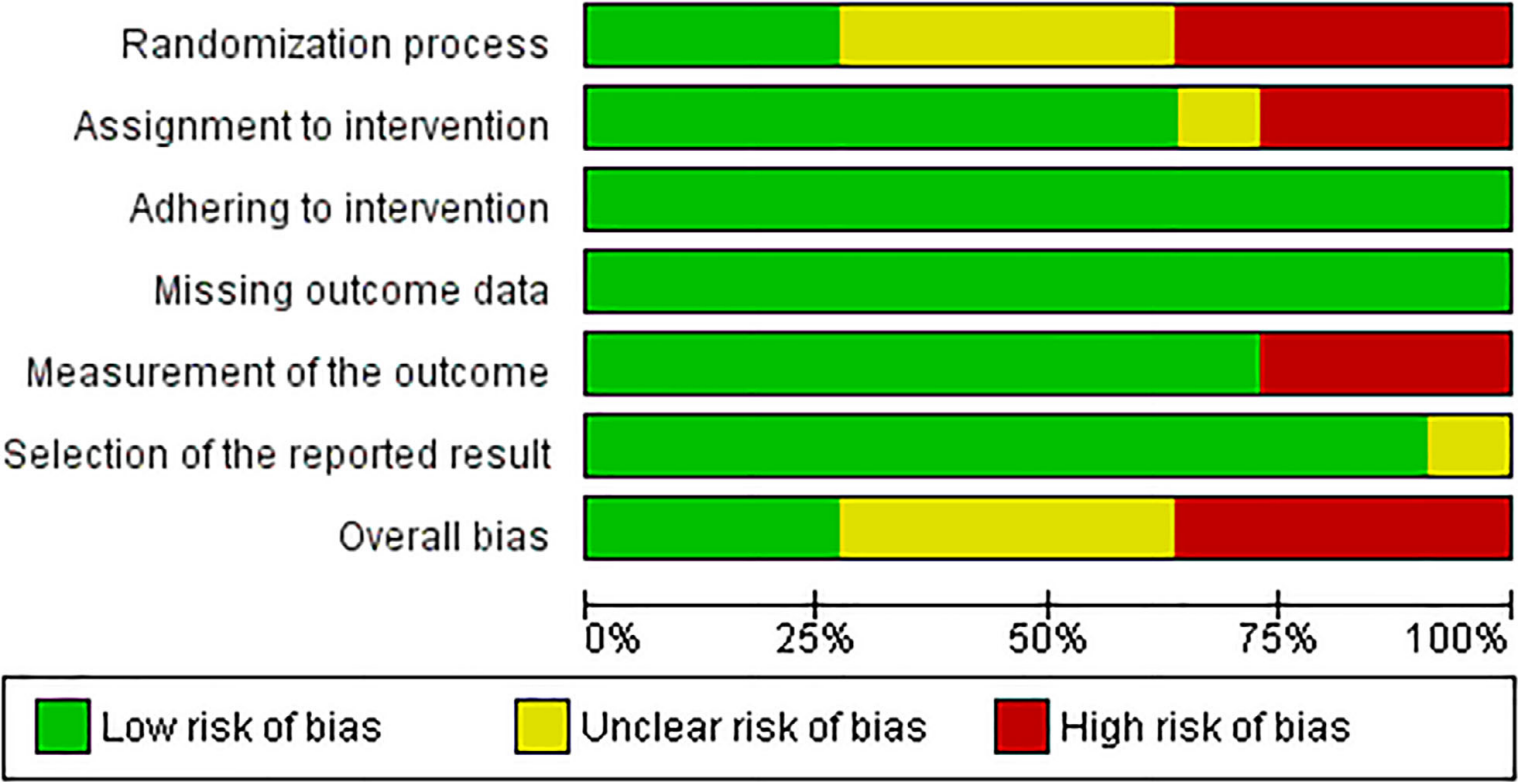
Risk of bias graph.

As assessed by the Cochrane Collaboration's RoB2 tool, four out of 11 studies (36.4%) showed a high risk of bias, four studies (36.4%) showed some concerns, and three studies (27.2%) had a low risk of bias. By domains, 36.4% showed high risk and 36.4% some concerns for randomization domain; 27.2% showed high risk and 9.1% some concerns for assignment to intervention; 27.2% showed high risk for measurement of the outcome; and 9.1% showed some concerns for selection of the reported result. No study showed risk of bias for the adhering to intervention and missing outcome data domains (Fig. 4, 5).

Overall, non‐RCTs scored as high risk of bias due to lack of randomization and blinding. There were also RCTs with some concerns in the “randomization process” domain because the randomization process was not entirely clear, as well as one Eteplirsen study which had a potential risk of bias in the “selection of the reported results” domain, by not reporting some TFTs and NSAA.

### Evidence assessment

Using the GRADE tool, Ataluren showed a high certainty of evidence for 6MWD, and moderate certainty for climb 4 stairs, descend 4 stairs, run 10 m, supine to stand and the NSAA. Drisapersen only had a high certainty for 6MWD, whereas results were moderate to very low uncertainty for the other TFTs and the NSAA. Eteplirsen showed a moderate certainty for 6MWD (Tables S2–S4).

### Meta‐analysis

Although there was a limited number of studies for each drug treatment in the different outcomes, due to the interest of the disease, a meta‐analysis with the existing data for the main outcomes (TFTs and NSAA) was conducted in order to show a pooled estimate to increase the currently available evidence (Figures S1–S6). Conversely, sensitivity analyses and subgroup analyses by baseline 6MWD were not examined due to the scarcity of studies.

The pooled mean difference for Ataluren in the 6MWD test was 18.3 m (95% CI: 1.0, 35.5) and for Drisapersen 21.5 m (95% CI: 4.7, 38.3). Additionally, for the others TFTs, the pooled mean difference for Ataluren was −1.7 sec (95% CI: −3.0, −0.4) for climb 4 stairs, −1.9 sec (95% CI: −3.2, −0.6) for descend 4 stairs and −1.6 sec. (95% CI: −3.1, −0.1) for run 10 m. However, effects were not statistically significant for Drisapersen. Finally, Drisapersen did not show a significant effect on NSAA.

Based on age at the start of the trial (<7 or ≥7 years old), a slightly higher effect was observed in younger children than in older ones for Drisapersen, in such a way that the pooled mean difference in younger children was 23.2 m (95% CI: −2.2, 48.5) in the Δ6MWD test, whereas it was 17.1 m (95% CI: −8.7, 42.9) among those aged ≥7 years. No data were obtained for Ataluren and Eteplirsen.

Heterogeneity was not important for 6MWD in both Ataluren and Drisapersen and the baseline age subgroups (I^2^ = 0.0%). Ataluren also had not important heterogeneity for the other outcomes (I^2^ = 0.0%), as well as Drisapersen in the tests “down 4 stairs” (I^2^ = 0.0%), “run 10 m” (I^2^ = 14.3%), and NSAA (I^2^ = 17.4%). However, Drisapersen showed moderate heterogeneity for the “up 4 stairs” test, and substantial heterogeneity for “supine to stand” (I^2^ = 71.6%). A publication bias analysis by funnel plot could not be performed due to the scarcity of studies. However, the existence of publication bias cannot be ruled out, based on: (1) there is one trial without published results (i.e., NCT02255552 for Eteplirsen), and (2) the 2013 Eteplirsen trial, showed risk of bias for “selection of the reported results” in which, for some reason, the authors did not publish the results of the tests they had previously committed to perform and publish.

## Discussion

This systematic review provides an overview of the evidence that supports a moderate effect of new drug treatments which aim to increase dystrophin synthesis in the progression of DMD. Our data confirm a limited but significant effect of Ataluren, Eteplirsen and Drisapersen on improving the 6MWD, the main outcome associated with DMD progression. Ataluren also showed a modest effect in the improvement of some of the others timed functional tests. Finally, all treatments tended to improve dystrophin expression, exon skipping (except Ataluren), respiratory function, and biochemical changes.

Although nonsense suppression therapy (Ataluren) and exon skipping (Eteplirsen, Drisapersen) are two different pharmacological strategies for different patients with DMD, for both strategies the target is the synthesis of a partially functional dystrophin. Thus, it seems reasonable to analyze and compare them together. It is estimated that approximately 25% of patients can benefit from the use of these pharmacological treatments. However, the potential omission of exons 8, 43, 44, 45, 46, 50, 52, 53, or 55 could increase the percentage of patients treated to 75%.^34^

Different dose–response effects were observed for Ataluren and Drisapersen. In in vitro studies, Ataluren showed a bell‐shaped concentration–response curve to suppress premature nonsense codons, with a dose‐dependent effect until a maximum, followed by a decrease.^35^ Bushby et al. confirmed this finding in humans. The maximum effect observed was obtained with 40 mg/kg per day (Δ6MWD = 31.3 m at 48 weeks), whereas the effect with 80 mg/kg per day was indistinguishable from the placebo (Δ6MWD=−0.7 m at 48 weeks). Interestingly, in the 80 mg/kg per day group, only patients who had plasma concentrations of ataluren similar to the plasma concentrations of patients given 40 mg/kg per day showed any effect. As for Drisapersen, McDonald et al. studied the effect of 3 mg/kg, obtaining results for lower than 6 mg/kg (Δ6MWD=−8.9 m vs. Δ6MWD = 27.1 m, respectively). It should be noted that the minimum clinically important differences in 6MWD at 12 months of treatment are set at approximately 30 m, although when physical function is low, a smaller effect on 6MWD can also improve health‐related quality of life.^36^ Goemans et al. determined that there was a dose‐dependent response until at least 6 mg/kg, with dystrophin levels showing an 8.2‐fold‐increase compared to the reference, corresponding to patients receiving 0.5 mg/kg. This study considered until 10 mg/kg, but the dose was not increased further. Moreover, in the extension phase in which all patients received 6 mg/kg, mild to moderate adverse events appeared in numerous patients, such as proteinuria, increased urinary α1‐microglobulin levels and local reactions, among others.^37^ Flanigan et al. also determined that adverse events were dose‐dependent, especially renal toxicity, inflammation and local reactions, confirming that the dose with the best benefit/risk profile was 6 mg/kg.^38^ Studies with Eteplirsen suggested a dose‐dependent response in the expression of dystrophin and dystrophin‐positive fibers up to 20 mg/kg, with a very good safety profile.^39^ However, no dose results higher than 50 mg/kg were used by Mendell et al. Although dystrophin expression levels were low, it remains unclear the threshold of dystrophin expression which results in an effect on the ability to ambulate and other motor skills can be obtained.^29^ In fact, the candidate patients for exon 44 skipping seem to take longer to lose the ambulation, coinciding with the fact that some of these patients skip exon 44 spontaneously.^40^ Therefore, it seems that Ataluren has a limited effect due to its action mechanism; however, antisense oligonucleotides have a dose–response effect, and could potentially be better if higher doses could be administered safely.

The benefits observed in 6MWD and others TFTs seem to be associated with a moderate increase in dystrophin expression, an increase in the presence of dystrophin‐positive fibers, the omission of exon 51 (for Eteplirsen and Drisapersen) and a decrease in CK. These associations suggest that these treatments could translate into slowing disease progression. Conversely, there were no positive effects on 6MWD and TFTs for Casimersen, although it seems that the increase in dystrophin and omission of exon 45 should be accompanied by clinical improvements. Moreover, the study by Komaki et al.^41^ with Viltolarsen, which omits exon 53, showed increases in dystrophin expression, therefore, it could also slow disease progression. The change in 6MWD seems especially relevant for Eteplirsen, however, this finding should be cautiously interpreted due to the low number of patients included, as well as the rapid deterioration of two of the patients who were excluded from the analysis. Eteplirsen has been conditionally approved by the FDA, causing a controversial discussion around its use^12^. For this reason, a new trial with a larger sample and longer duration, the PROMOVI trial (NCT02255552), has been commissioned to the *Sarepta Therapeutics* company.

The analyses by age subgroups, <7 or ≥7 years old, together with a modest overall effect, suggest that Drisapersen has a limited effect on the 6MWD test, backed by lower dystrophin expression, dystrophin‐positive fibers, and exon 51 skipping. However, Drisapersen has more adverse events than Eteplirsen, being one of the reasons why the FDA rejected its approval, and its development research for commercial use has been no longer pursued. Although it is not clear, this could be due to the different chemical structure of Drisapersen compared to Eteplirsen. Drisapersen is a 2′‐O‐methyl phosphorothionate antisense oligonucleotide (2OMeAO), whereas Eteplirsen is a phosphorodiamidate morpholino oligomer (PMO).^42^ PMOs have a neutral charge, they are also more soluble, which allows a greater dosage of the drug to be administered, and they do not present immune responses with their administration. Conversely, Ataluren does seem to have a greater effect in children >7 years with a lower 6MWD baseline.

The effect of these drug treatments on the secondary outcomes also indicates some effect in DMD. Eteplirsen improved FVC and MIP, and Ataluren improved FVC,^43^ whereas Drisapersen did not improve respiratory function. There was also a decrease in CK and LDH levels for Drisapersen in the trial of Goemans et al., although in the trial of McDonald et al. no change was reported. This may be due to a lower inflammatory state, suggesting some improvements. However, the discordance between these two studies, along with the fact that CK decreases over time in patients with DMD due to progressive decrease in muscle mass, questions the effect of Drisapersen on CK. No data were reported for cardiac function. However, this outcome should be a critical endpoint in clinical trials, since mortality in these patients usually occurs due to heart failure.^44^

DMD has a high direct medical and nonmedical financial cost, as well as indirect financial costs. Furthermore, some authors have shown that the quality of life of these patients decreases markedly over time.^45^ The limited available evidence suggests that Ataluren is associated with a better state of health and utility.^46^ However, the cost‐utility relationship of these new therapies is hardly studied, nor what effect does it have on the quality of life of patients, and it is something that should be investigated.

There are some important limitations in this systematic review. First, the scarcity of controlled trials assessing the effect of these pharmacological treatments on the progression of DMD in humans limits the available evidence. Although the results of the included studies as well as the results of our systematic review support that Ataluren and Eteplirsen have a good safety profile and moderate effectiveness, drug agencies usually require a high level of evidence for the approval of new drug therapies. Moreover, in the short‐medium term it is not likely that the evidence will increase substantially as DMD is a rare disease and research is inevitably limited. Second, the long‐term effect of these drugs has not been established, with 1‐year trials being common, since it is assumed that differences between intervention and control groups persist after this time period. Third, sensitivity and subgroup analyses from pooled estimates could not be performed as was reported in the published protocol due to the small number of studies included. Fourth, a publication bias analysis was also not possible due to the small number of studies.^47^ This is an important limitation, as small or no‐effect studies tend not to be published. Fifth, some of the data from the Ataluren trials come from post hoc analyses, which may overestimate the effect on the main outcomes. Sixth, some studies have used matched historical cohorts as comparison group, especially with Eteplirsen, and therefore de provided data, since they did not come from a randomized trial, could distort the final result. Finally, the risk of bias of some included studies could threaten the validity of the results and the strength of the available evidence.

## Conclusions

There is limited evidence of the effectiveness of new treatments for DMD. Our findings seem to confirm a moderate benefit for all drug treatments in the progression of the disease, improving its phenotype. Thus, our pooled estimates suggest that Ataluren and Drisapersen improve 6MWD, and Ataluren improves others TFTs. Eteplirsen may also improve respiratory function. Although we suggest that early access to these treatments may modestly delay the loss of ambulation ability and motor impairment in the medium term, they are very expensive and there are not consistent evidence about their long‐term effects. Therefore, long‐term trials are needed to verify the real effect of these pharmacological treatments, as well as studies to verify the improvement in the quality of life in these patients and cost‐utility studies.

## Conflict of Interest

None declared.

## Author Contribution

CP‐M designed, coordinated, conducted, and wrote the study. IC‐R, CA‐B, AE‐M, and DP‐C gave statistical and epidemiological support. CA‐B coordinated and conducted the study. VM‐V was the principal investigator and guarantor.

## Data Sharing Statement

Not applicable.

## Supporting information


**Table S1.** Excluded studies with reasons.
**Table S2.** Grades of Recommendation, Assessment, Development, and Evaluation of ataluren.
**Table S3.** Grades of Recommendation, Assessment, Development, and Evaluation of Eteplirsen.
**Table S4.** Grades of Recommendation, Assessment, Development, and Evaluation of Drisapersen.
**Figure S1.** Forest plot for mean difference in 6‐minute walking distance test by pharmacological treatment.
**Figure S2.** Forest plot for mean difference in up 4 stairs test by pharmacological treatment.
**Figure S3.** Forest plot for mean difference in down 4 stairs test by pharmacological treatment.
**Figure S4.** Forest plot for mean difference in run 10 m test by pharmacological treatment.
**Figure S5.** Forest plot for mean difference in supine to stand test for Drisapersen.
**Figure S6.** Forest plot for mean difference in the North Star Ambulatory Assessment for Drisapersen.
**Appendix S1.** Search strategy.
