# Supplementary material for: Restorative treatments of dystrophin expression in Duchenne muscular dystrophy: A systematic review
Source: Ann Clin Transl Neurol. 2020 Aug 10;7(9):1738–52. doi: 10.1002/acn3.51149 (PMC7480922; doi:10.1002/acn3.51149)
Supplement: Supplementary file 1 — Table S1. Excluded studies with reasons. Table S2. Grades of Recommendation, Assessment, Development, and Evaluation of ataluren. Table S3. Grades of Recommendation, Assessment, Development, and Evaluation of Eteplirsen. Table S4. Grades of Recommendation, Assessment, Development, and Evaluation of Drisapersen. Figure S1. Forest plot for mean difference in 6‐minute walking distance test by pharmacological treatment. Figure S2. Forest plot for mean difference in up 4 stairs test by pharmacological treatment. Figure S3. Forest plot for mean difference in down 4 stairs test by pharmacological treatment. Figure S4. Forest plot for mean difference in run 10 m test by pharmacological treatment. Figure S5. Forest plot for mean difference in supine to stand test for Drisapersen. Figure S6. Forest plot for mean difference in the North Star Ambulatory Assessment for Drisapersen. Appendix S1. Search strategy. [file ACN3-7-1738-s001.docx]

**Table S1.** Excluded studies with reasons

| Reference | Drug | Reason for exclusion |
| --- | --- | --- |
| Wagner KR, et al. (2001)^1^ | Gentamicin | Not controlled |
| Finkel RS, et al. (2013)^2^ | Ataluren | Not controlled |
| NCT00759876 | Ataluren | Without results. Not controlled |
| NCT01009294/EudraCT:2009-013169-24 | Ataluren | Without results. Not controlled |
| NCT00847379 | Ataluren | Without results. Not controlled |
| NCT02819557 | Ataluren | Without results. Not controlled |
| NCT03648827 | Ataluren | Active. Not controlled |
| NCT01247207 | Ataluren | Enrolling. Not controlled |
| NCT03179631 | Ataluren | Recruiting |
| NCT01557400/EudraCT:2011-004853-18 | Ataluren | Without results. Not controlled |
| NCT02090959/EudraCT:2013-005489-20 | Ataluren | Without results. Not controlled |
| Kinali M, et al. (2009)^3^ | Eteplirsen | Not controlled |
| Cirak S, et al. (2011)^4^ | Eteplirsen | Not controlled |
| NCT03985878 | Eteplirsen | Not yet recruiting. Not controlled |
| NCT02286947/EudraCT:2016-005024-28 | Eteplirsen | Without results. Not controlled |
| NCT03992430 | Eteplirsen | Not yet recruiting |
| NCT02255552/EudraCT:2016-005002-19 | Eteplirsen | Without results |
| NCT03218995 | Eteplirsen | Recruiting. Not controlled |
| NCT02420379/EudraCT:2016-005023-92 | Eteplirsen | Without results. Not controlled |
| Van Deutekom JC, et al. (2007)^5^ | Drisapersen | Not controlled |
| Goemans NM, et al. (2011)^6^ | Drisapersen | Not controlled |
| Flanigan KM et al (2014)^7^ | Drisapersen | No outcomes of interest |
| Goemans NM, et al. (2016)^8^ | Drisapersen | Not controlled |
| Goemans NM, et al. (2017)^9^ | Drisapersen | No outcomes of interest |
| NCT01890798 | Drisapersen | Withdrawn. Not controlled |
| NCT01480245/EudraCT:2011-001266-17 | Drisapersen | Without results. Not controlled |
| NCT03532542/EudraCT:2017-004625-32 | Casimersen, golodirsen | Enrolling |
| NCT02310906 | Golodirsen | Without results |
| Komaki H et al (2018)^10^ | Viltolarsen | Not controlled |
| NCT04060199 | Viltolarsen | Not yet recruiting |
| NCT03167255 | Viltolarsen | Active |
| NCT02740972 | Viltolarsen | Not controlled |

Supplementary references

1. Wagner KR, Hamed S, Hadley DW, et al. Gentamicin treatment of Duchenne and Becker muscular dystrophy due to nonsense mutations. Ann. Neurol. 2001;49(6):706–711.
2. Finkel RS, Flanigan KM, Wong B, et al. Phase 2a study of ataluren-mediated dystrophin production in patients with nonsense mutation Duchenne muscular dystrophy. PLoS One 2013;8(12)
3. Kinali M, Arechavala-Gomeza V, Feng L, et al. Local restoration of dystrophin expression with the morpholino oligomer AVI-4658 in Duchenne muscular dystrophy: a single-blind, placebo-controlled, dose-escalation, proof-of-concept study. Lancet Neurol. 2009;8(10):918–928.
4. Cirak S, Arechavala-Gomeza V, Guglieri M, et al. Exon skipping and dystrophin restoration in patients with Duchenne muscular dystrophy after systemic phosphorodiamidate morpholino oligomer treatment: An open-label, phase 2, dose-escalation study. Lancet 2011;378(9791):595–605.
5. van Deutekom JC, Janson AA, Ginjaar IB, et al. Local Dystrophin Restoration with Antisense Oligonucleotide PRO051 [Internet]. N. Engl. J. Med. 2007;357(26):2677–2686.
6. Goemans NM, Tulinius M, Van Den Akker JT, et al. Systemic administration of PRO051 in Duchenne’s muscular dystrophy. N. Engl. J. Med. 2011;364(16):1513–1522.
7. Flanigan KM, Voit T, Rosales XQ, et al. Pharmacokinetics and safety of single doses of drisapersen in non-ambulant subjects with Duchenne muscular dystrophy: Results of a double-blind randomized clinical trial. Neuromuscul. Disord. 2014;24(1):16–24.
8. Goemans NM, Tulinius M, van den Hauwe M, et al. Long-Term Efficacy, Safety, and Pharmacokinetics of Drisapersen in Duchenne Muscular Dystrophy: Results from an Open-Label Extension Study [Internet]. PLoS One 2016;11(9):e0161955.
9. Goemans N, Tulinius M, Kroksmark A-K, et al. Comparison of ambulatory capacity and disease progression of Duchenne muscular dystrophy subjects enrolled in the drisapersen DMD114673 study with a matched natural history cohort of subjects on daily corticosteroids [Internet]. Neuromuscul. Disord. 2017;27(3):203–213.
10. Komaki H, Nagata T, Saito T, et al. Systemic administration of the antisense oligonucleotide NS-065/NCNP-01 for skipping of exon 53 in patients with Duchenne muscular dystrophy. Sci. Transl. Med. 2018;10(437)

**Table S2.** Grades of Recommendation, Assessment, Development, and Evaluation of Ataluren

| Certainty assessment | | | | | | | Nº of patients | | Effect | | Certainty | Importance |
| --- | --- | --- | --- | --- | --- | --- | --- | --- | --- | --- | --- | --- |
| Nº of studies | **Study design** | **Risk of bias** | **Inconsistency** | **Indirectness** | **Imprecision** | **Other considerations** | **Ataluren** | **No intervention** | **Relative (95% CI)** | **Absolute (95% CI)** |  |  |
| Six Minute Walking Distance (assessed with: meters) | | | | | | | | | | | | |
| 2 | RCT | not serious | not serious | not serious | not serious | none | 172 | 172 | - | see comment | HIGH | CRITICAL |
| TFT - Climb 4 stairs (assessed with: seconds) | | | | | | | | | | | | |
| 2 | RCT | not serious | not serious | not serious | serious ^a^ | none | 172 | 172 | - | see comment | MODERATE | IMPORTANT |
| TFT - Descend 4 stairs (assessed with: seconds) | | | | | | | | | | | | |
| 2 | RCT | not serious | not serious | not serious | serious ^a^ | none | 172 | 172 | - | see comment | MODERATE | IMPORTANT |
| TFT - Run 10 m (assessed with: meters) | | | | | | | | | | | | |
| 2 | RCT | not serious | not serious | not serious | serious ^a^ | none | 172 | 172 | - | see comment | MODERATE | IMPORTANT |
| TFT - Supine to stand (assessed with: seconds) | | | | | | | | | | | | |
| 1 | RCT | not serious | not serious | not serious | serious ^a^ | none | 57 | 57 | - | see comment | MODERATE | IMPORTANT |
| The North Star Ambulatory Assessment | | | | | | | | | | | | |
| 1 | RCT | not serious | not serious | not serious | serious ^a^ | none | 115 | 115 | - | see comment | MODERATE | IMPORTANT |

**CI:** Confidence interval, a: Large confidence intervals

**Table S3.** Grades of Recommendation, Assessment, Development, and Evaluation of Eteplirsen

| Certainty assessment | | | | | | | Nº of patients | | Effect | | Certainty | Importance |
| --- | --- | --- | --- | --- | --- | --- | --- | --- | --- | --- | --- | --- |
| Nº of studies | **Study design** | **Risk of bias** | **Inconsistency** | **Indirectness** | **Imprecision** | **Other considerations** | **Eteplirsen** | **No intervention** | **Relative (95% CI)** | **Absolute (95% CI)** |  |  |
| Six Minute Walking Distance (assessed with: Meters) | | | | | | | | | | | | |
| 1 | RCT | serious ^a^ | not serious | not serious | serious ^b^ | strong association | 6 | 4 | - | see comment | MODERATE | CRITICAL |

CI: Confidence interval, a: Some concerns, b: Large confidence interval. Estimation from p.

**Table S4.** Grades of Recommendation, Assessment, Development, and Evaluation of Drisapersen

| Certainty assessment | | | | | | | Nº of patients | | Effect | | Certainty | Importance |
| --- | --- | --- | --- | --- | --- | --- | --- | --- | --- | --- | --- | --- |
| Nº of studies | **Study design** | **Risk of bias** | **Inconsistency** | **Indirectness** | **Imprecision** | **Other considerations** | **Drisapersen** | **No intervention** | **Relative (95% CI)** | **Absolute (95% CI)** |  |  |
| 6 - Minute Walking Distance (assessed with: Meters) | | | | | | | | | | | | |
| 3 | RCT | not serious | not serious | not serious | not serious | none | 161 | 95 | - | see comment | HIGH | CRITICAL |
| TFT - Climb 4 stairs (assessed with: seconds) | | | | | | | | | | | | |
| 3 | RCT | not serious | serious ^a^ | not serious | serious ^b^ | none | 161 | 95 | - | see comment | LOW | IMPORTANT |
| TFT - Descend 4 stairs (assessed with: seconds) | | | | | | | | | | | | |
| 3 | RCT | not serious | not serious | not serious | serious ^b^ | none | 161 | 95 | - | see comment | MODERATE | IMPORTANT |
| TFT - Run 10 meters (assessed with: seconds) | | | | | | | | | | | | |
| 3 | RCT | not serious | serious | not serious | serious ^b^ | none | 161 | 95 | - | see comment | LOW | IMPORTANT |
| TFT - Supine to stand (assessed with: Seconds) | | | | | | | | | | | | |
| 2 | RCT | not serious | very serious ^a^ | not serious | serious ^b^ | none | 36 | 34 | - | see comment | VERY LOW | IMPORTANT |
| The North Star Ambulatory Assessment | | | | | | | | | | | | |
| 3 | RCT | not serious | not serious | not serious | serious ^b^ | none | 161 | 95 | - | see comment | MODERATE | IMPORTANT |

CI: Confidence interval, a: non-uniform direction of effect, b: Large confidence intervals

**Figure S1.** Forest plot for mean difference in six-minute walking distance test by pharmacological treatment


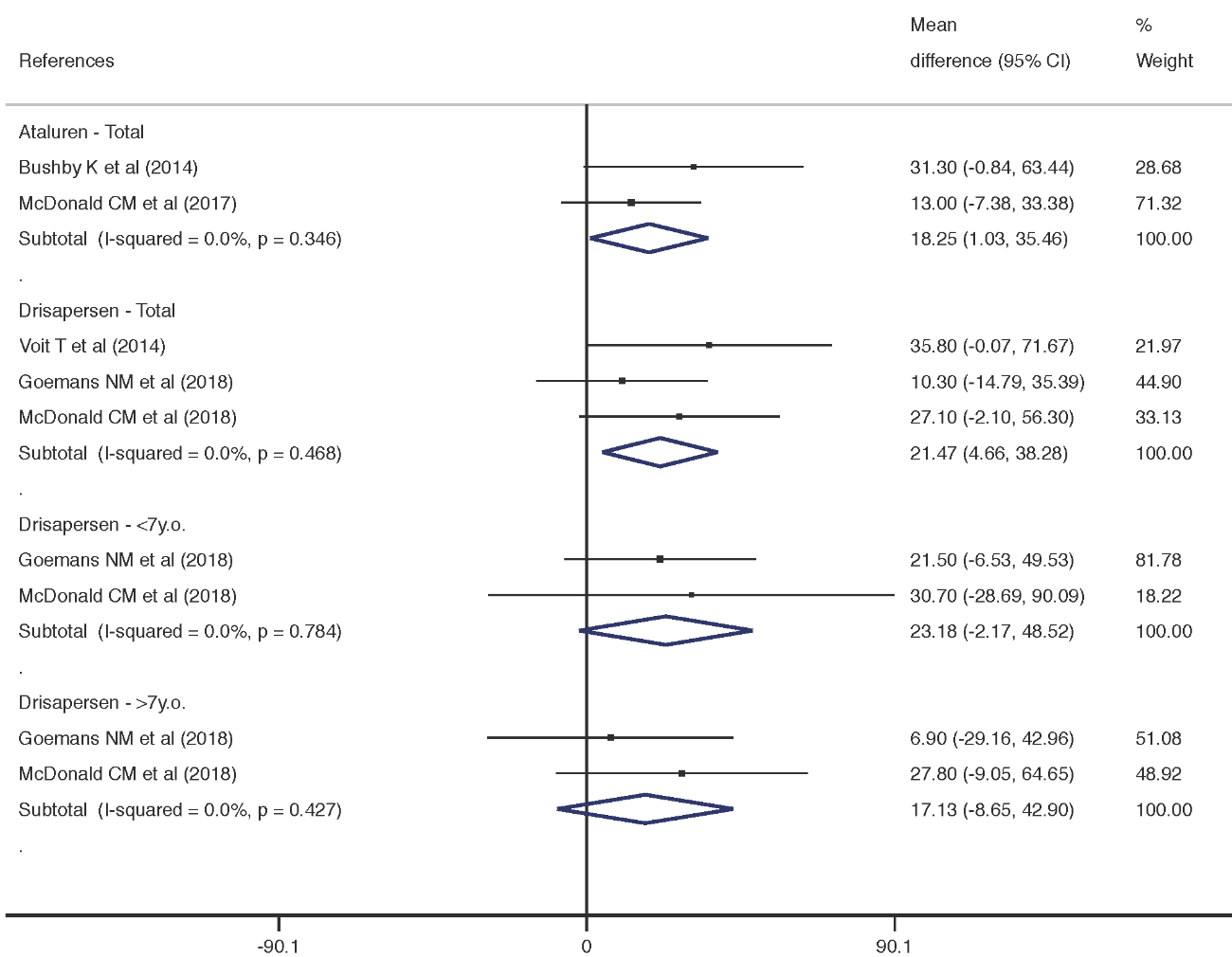


Positive effect indicates effect in favor of the intervention group, while negative effect indicates effect in favor of the control group.

**Figure S2.** Forest plot for mean difference in up 4 stairs test by pharmacological treatment


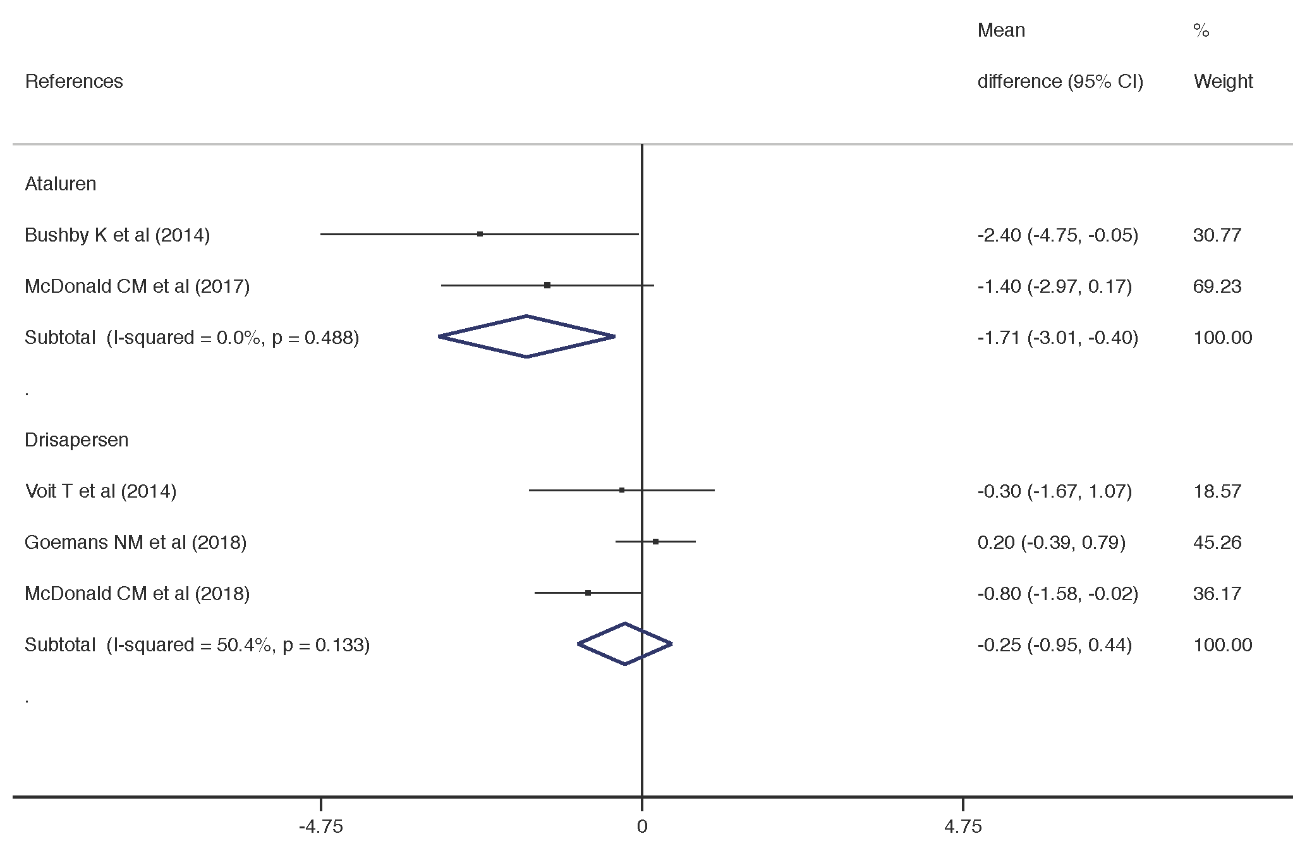


Negative effect indicates effect in favor of the intervention group, while positive effect indicates effect in favor of the control group.

**Figure S3.** Forest plot for mean difference in down 4 stairs test by pharmacological treatment


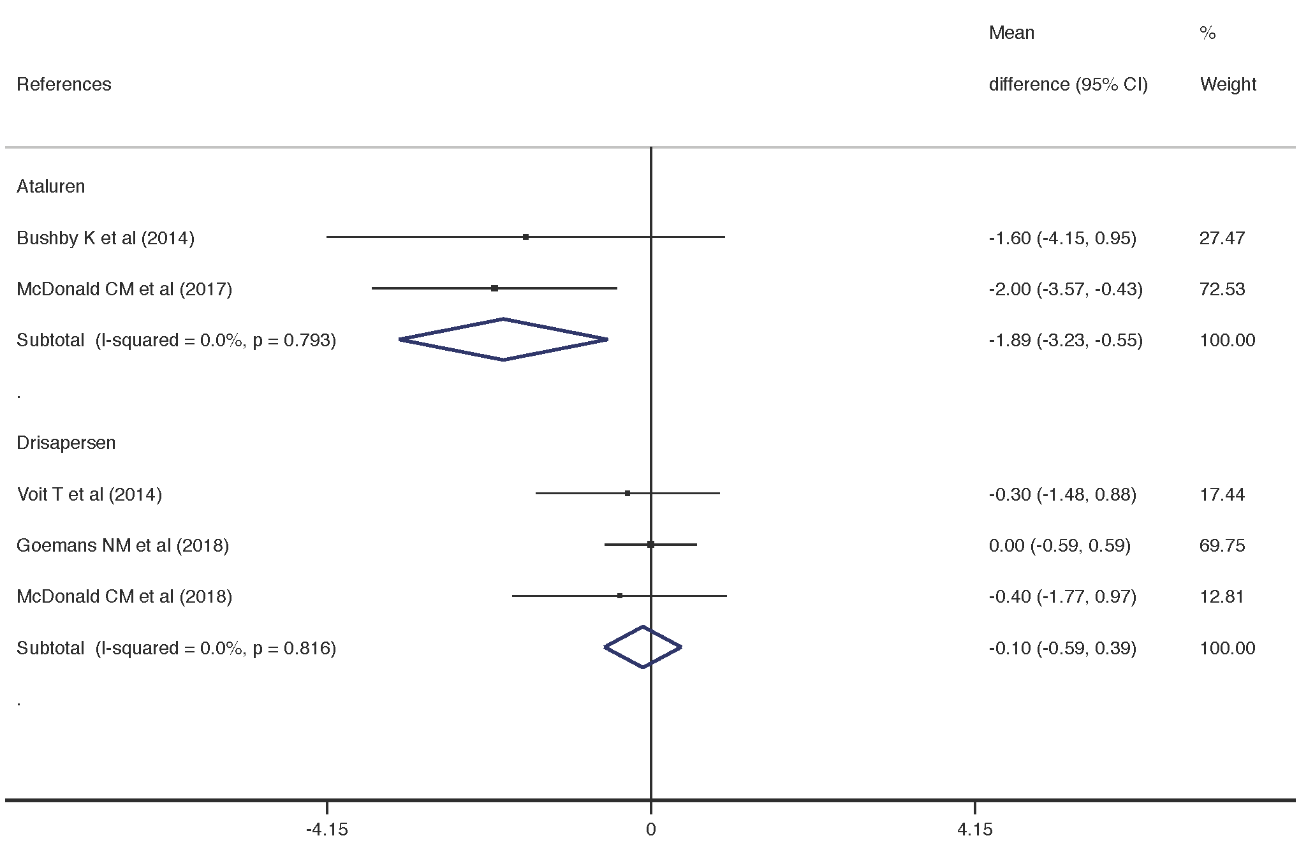


Negative effect indicates effect in favor of the intervention group, while positive effect indicates effect in favor of the control group.

**Figure S4.** Forest plot for mean difference in run 10 meters test by pharmacological treatment


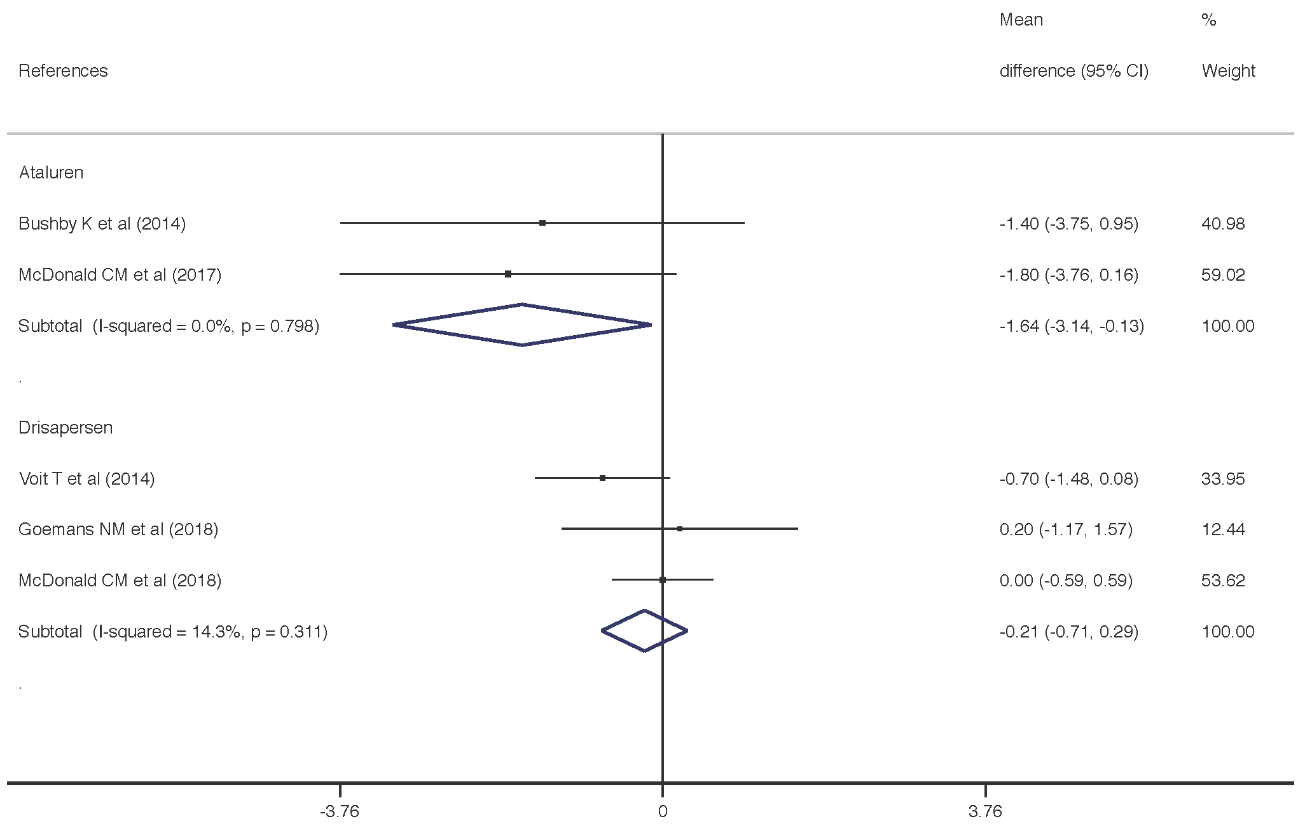


Negative effect indicates effect in favor of the intervention group, while positive effect indicates effect in favor of the control group.

**Figure S5.** Forest plot for mean difference in supine to stand test for Drisapersen


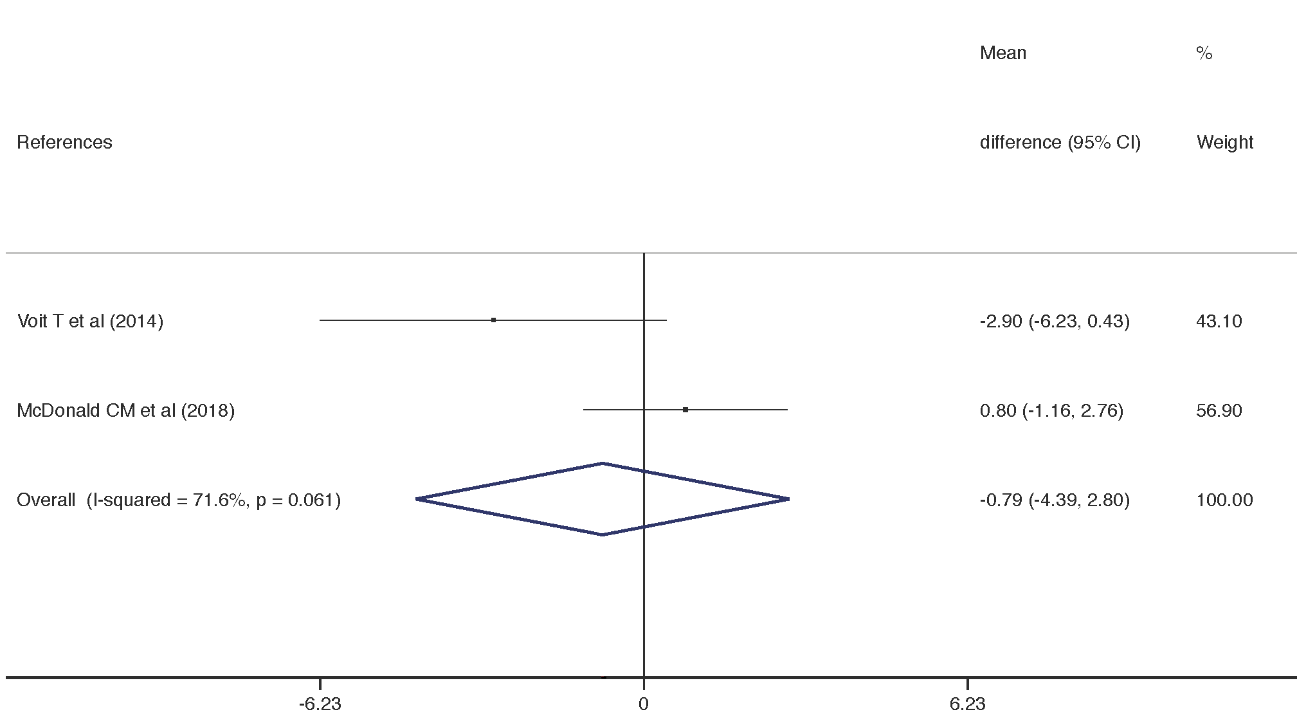


Negative effect indicates effect in favor of the intervention group, while positive effect indicates effect in favor of the control group.

**Figure S6.** Forest plot for mean difference in the north star ambulatory assessment for Drisapersen


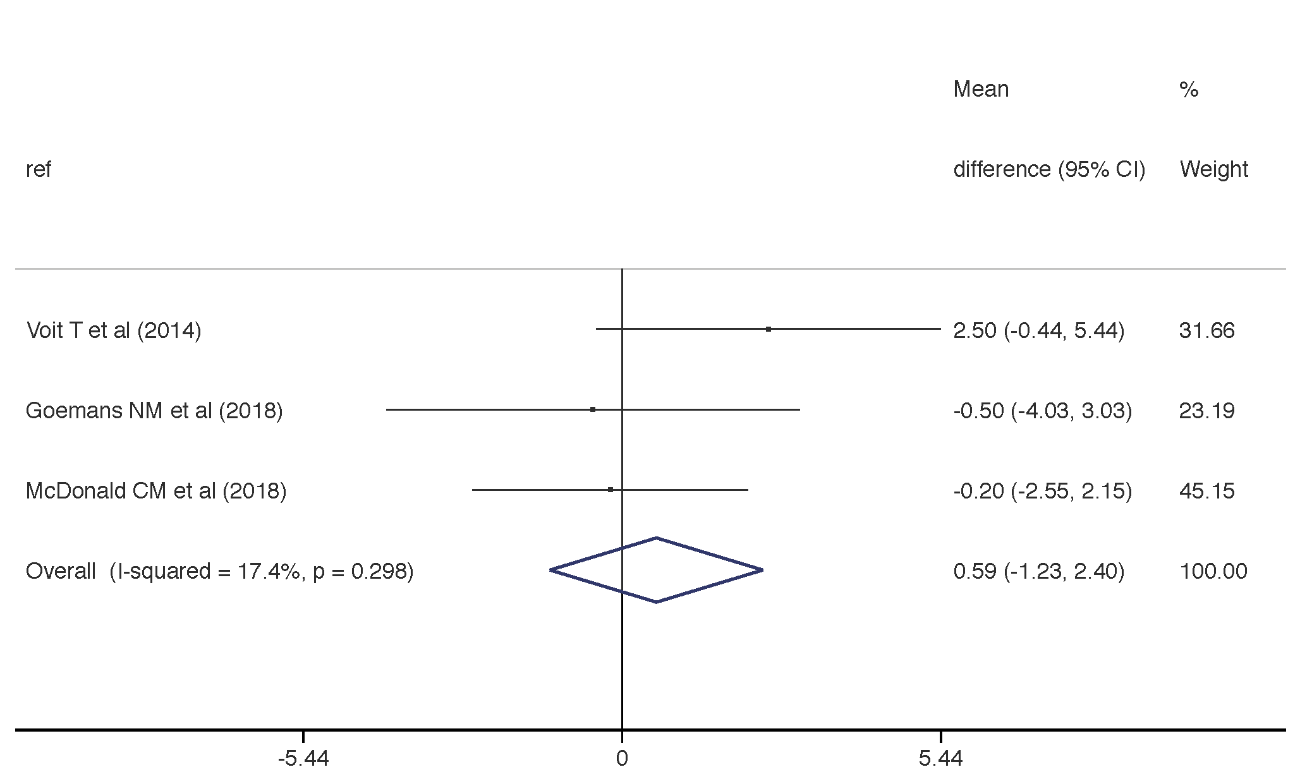


Positive effect indicates effect in favor of the intervention group, while negative effect indicates effect in favor of the control group.

**Appendix S1.** Search Strategy

- Medline, EMBASE, Web of Science:

(“dystrophy” OR “duchenne” OR “dmd” OR “dystrophinopathy”) AND (“gentamicin” OR “ataluren” OR “ptc124” OR “eteplirsen” OR “avi-4658” OR “drisapersen” OR “pro051” OR “golodirsen” OR “casimersen” OR “viltolarsen” OR “antisense oligonucleotides” OR “exon skipping” OR “nonsense readthrough” OR “stop codon suppression” OR “translational readthrough” OR “nonsense suppression”) AND (“trial” OR “randomized clinical trial” OR “randomized controlled trial” OR “clinical trial”)

- NCT Trials:

Condition or disease: Duchenne; Other terms: gentamicin

Condition or disease: Duchenne; Other terms: ataluren

Condition or disease: Duchenne; Other terms: eteplirsen

Condition or disease: Duchenne; Other terms: drisapersen

Condition or disease: Duchenne; Other terms: golodirsen

Condition or disease: Duchenne; Other terms: casimersen

Condition or disease: Duchenne; Other terms: viltolarsen

Condition or disease: Duchenne; Other terms: gentamicin

Condition or disease: Duchenne; Other terms: gentamicin

- EudraCT:

(“dystrophy” OR “duchenne” OR “dmd” OR “dystrophinopathy”) AND (“gentamicin” OR “ataluren” OR “ptc124” OR “eteplirsen” OR “avi-4658” OR “drisapersen” OR “pro051” OR “golodirsen” OR “casimersen” OR “viltolarsen”)

- Other databases

Open search (not specified).
